# Supplementary material for: Long-Term Clinical Outcome and Prognostic Factors of Children and Adolescents with Localized Rhabdomyosarcoma Treated on the CWS-2002P Protocol
Source: Cancers (Basel). 2022 Feb 11;14(4):899. doi: 10.3390/cancers14040899 (PMC8870315; doi:10.3390/cancers14040899)
Supplement: Supplementary file 1 [file cancers-14-00899-s001.zip › cancers-1580159-supplementary.pdf]

Supplementary material

Contents

Table S1 Factors included in the risk stratification in the CWS studies -81, 86,-91,-96 and -2002P

Table S2 Patient characteristics in the High Risk Group by therapy arm: maintenance vs. no maintenance therapy

Table S3 Characteristics of patients with second malignancy.

Table S4 Univariate analysis

Table S5 Multivariate analysis

Table S6 Univariate analysis considering individual tumour sites and fusion status

Table S1
Factors included in the risk stratification in the CWS studies -81, 86,-91,-96 and -2002P

	IRS-Group	Response	Primary site	N-Status	Histology	T-Status	Age	Tumor size
CWS-81	+	-	-	-	-	-	-	-
CWS-86	+	+	-	-	-	-	-	-
CWS-91	+	+	+	+	+	-	-	-
CWS-96	+	+	+	+	+	+	-	-
CWS-2002P	+	+	+	+	+	-	+	+

Table S2. Patient characteristics in the High Risk Group by therapy arm: maintenance vs. no maintenance therapy.

[1]not tested

Variable	Total No.	Total %	Maintenance No.	Maintenance %	No Maintenance No.	No Maintenance %	P value*
	204	100	155	100	49	100	
**Gender**							0·93
male	114	56	86	55	28	57	
female	90	44	69	45	21	43	
**Age**							0·4
≤10 yrs	150	74	111	72	39	80	
>10yrs	90	26	44	28	10	20	
**Histology**							0·06
aRMS fusion positiv	33	15	30	19	3	6	
aRMS fusion negativ	7	3	6	4	1	2	
aRMS fusion ns/na[1]	14	7	9	6	5	10	
non aRMS	150	70	110	71	40	82	
**Primary tumour site**							0·94
EXT	29	14	20	13	9	18	
HN-nPM	20	10	12	10	5	10	
HN-PM	64	31	50	32	14	29	
ORBITA	3	1	3	2	-	-	
GU-BP	29	14	22	14	7	14	
GU-nBP	6	3	4	3	2	4	
OTHER	52	25	40	26	12	24	
not specified	1	0,49	1	0,64	-	-	
**Primary tumoursize**							0·58
≤5cm	58	28	46	30	12	24	
>5cm	135	66	101	65	34	69	
not specified	11	5	8	5	3	6	
**IRSG**							0·29
I	5	2	3	2	2	4	
II	23	11	20	13	3	6	
III	176	86	132	85	44	90	
Tumor invasiveness							0·48
T1	70	34	50	32	20	41	
T2	115	56	89	57	26	53	
TX	18	9	16	10	3	6	
**Regional lymph nodes**							0·64
N0	147	72	112	72	35	71	
N1	38	19	29	19	9	18	
Nx	19	9	14	9	5	11	

*Fishers exact test

Table S3
Characteristics of patients with second malignancy

Gender	Risk-Group	Primary Histology	PT-Site	RTX-received	RTX-Dose/Gy	SM-Diagnosis	Time to SM/yrs	SM-Site
male	HR	RMA	EXT-upper	ukn		ALL	1·97	na
female	SR	RME	HN-nPM	yes	44·8	AML / MDS	3·44	na
female	SR	RME	Orbita left	yes	41·4	Plexuspapilloma	2·74	CNS
female	SR	RME	Orbita right	yes	45	Brain tumour nos	1·12	CNS
male	SR	RME	HN-PM	yes	54	Ewing Sa.	5·72	Skull
male	SR	RME	GU-nBP paratesticular	no	0	Glioblastoma	3·67	CNS
female	HR	RME	Other-pelvis	yes	50·4	Glioblastoma	7·66	CNS
male	SR	RME	Orbita right	yes	45	Glioma low malign.	7·46	CNS
female	SR	RME	Orbita left	yes	45	LGG	3·81	CNS
male	SR	RME	GU-BP	no	0	M. Hodgkin	9·41	
female	SR	RME	GU-BP-	no	0	Medulloepithelioma	2·76	CNS
female	SR	RME	Orbita	yes	50·4	Meningeoma	11·97	CNS
male	HR	RMA	EXT-upper	yes	44·8	MPNST	10·07	ukn
male	HR	RME	OTHER abdomen	no	0	NBS	0·01	pelvis
female	HR	RME	OTHER thoracic wall	no	0	NBS	0·36	Thorax, abdomen
male	HR	RME	OTHER-thorax	no	0	Osteosarcoma	8·86	EXTR-Femur
male	HR	RME	EXT thigh	yes	45	Osteosarcoma	10·51	EXTR-Femur
female	SR	RME	OTHER-paravertebral	yes	54	Osteosarcoma	5.·4	Pelvis
male	HR	RME	HN-PM	yes	54	Osteosarcoma	6·05	Mandibula
male	SR	RME	HN-nPM	yes	50·4	Epithelial Ca.	7·93	Tongue
female	SR	RME	Orbita right	no	0	pNET	4·11	ukn
male	HR	RMA	HN-nPM	yes	45	Tyroid Ca.	7·10	Thyroid gland

PT: primary tumour site; SM: second malignancy; HR: High Risk; SR: Standard Risk; RT: Radiotherapy

Table S4

Univariate analysis

Variable	Category	n [1]	HR EFS	95% CI	p(HR)	HR OS	95% CI	p(HR)
**Age**	<10	302	1·0			1·0		
	≥10	107	1·31	0·87-1·95	0·17	1·43	90·0-2·26	0·13
**Tumor Size**	≤5cm	213	1·0			1·0		
	>5cm	196	1·85	1·28-2·68	0·001*	2·37	1·50-3·73	0·0002*
**IRS Stage**	I	52	1·0			1·0		
	II	63	1·24	0·44-3·48	0·68	1·0	0·27-3·72	1.0
	III	294	3·50	1·53-7·97	0·003*	3·58	1·31-9·79	0·01*
**Histology**	favourable	335	1·0			1·0		
	unfavourable	74	1·84	1·22-2·77	0·003*	2·46	1·55-3·90	0·0001*
**Primary site**	favourable	162	1·0			1·0		
	unfavorable	247	1·75	1·17-2·61	0·006*	3·39	1·94-5·92	1·9e-05*
**T Status**	T1	208	1·0			1·0		
	T2	201	2·13	1·46-3·11	8·4e-05*	3·01	1·87-4·84	5·2e-06*
**N Status**	N0	347	1·0			1·0		
	N1	62	2·40	1·58-3·63	3·8e-05*	3·12	1·96-4·97	1·6e-06*

[1]For 409 patients all data for univariate analysis were available

*significant

Table S5

Multivariate analysis

Variable	Category	n [1]	HR EFS	95% CI	p(HR)	HR OS	95% CI	p(HR)
**Age, years**								
	<10	302	1·0			1·0		
	≥10	107	1·22	0·80-1·80	0·35	1·22	0·75-1·98	0·43
**Tumor Size cm**								
	≤5	213	1·0			1·0		
	>5	196	1·38	0·92-2·08	0·12	1·43	0·88-2·31	0·15
**IRS Group**								
	I	52	1·0			1·0		
	II	63	1·24	0·43-3·57	0·68	0·70	0·18-2·76	0·61
	III	294	2·87	1·17-7·01	0·02*	1·43	0·46-4·51	0·54
**Histology**								
	favourable	335	1·0			1·0		
	unfavourable	74	1·50	0·96-2·35	0·07	1·79	1·08-2·97	0·002*
**Tumor Site**								
	favourable	162	1·0			1·0		
	unfavourable	247	0·83	0·52-1·34	0·45	1·75	0·90-3·42	0·10
**T-Status**								
	T1	208	1·0			1·0		
	T2	201	1·55	1·02-2·36	0·02*	1·81	1·08-3·01	0·02*
**Nodal Status**								
	N0	347	1·0			1·0		
	N1	62	1·57	1·01-2·45	0·04*	1·82	1·1-3·0	0·02*

[1] for 409 patients all data was available

*siginificant

Table S6. Univariate analysis considering individual tumour site and fusion status

Variable	Category	N[1] 409	5 year survival EFS	95% CI	p	OS	95% CI	p
**Age, years**								
	<10	302	74·6	69·8-79·7	0.17	82·1	77·8-83·7	0·13
	≤10	107	67·2	58·7-76·9		74·7	66·8-83·7	
**Gender**								
	female	164	69·7	63·0-77·2	0·52	78·8	72·7-85·4	0·90
	male	245	74·7	69·4-80·4		81·2	76·4-86·3	
**Tumor Size**	≤5cm	223	79·4	74·2-85·0	0·002*	87·1	82·7-91·7	0·0003*
	>5cm	206	65·7	59·6-72·6		72·2	66·3-78·7	
**IRS Stage**	I	52	89·6	81·4-98·7	9·2e-05*	93·8	87·3-100·0	0·0006*
	II	63	85·6	77·4-94·8		91·9	85·3-99·0	
	III	294	67·0	61·8-72·6		75·2	70·5-80·5	
**Histology**								
Group (fusion status	favourable	335	75·1	71·2-80·5	0·003*	83·7	79·8-87·9	7·2e-05*
Unconsidered)	unfavourable	74	59·3	49·1-71·7		64·4	54·3-76·4	
Fusion status	aRMS-positive	50	54·0	41·8-69·7	0·002*	61·8	49·7-76·9	4·3e-05*
considered	aRMS-negative	6	100·0			100·0		
	Fusion status – ukn.	18	61·1	42·3-88·3		59·1	39·6-88·1	
	non aRMS	335	75·7	71·2-80·5		83·7	79·8-87·8	
**Tumor Site**								
	OTHER	62	60·8	49·7-74·4	0·006*	67·1	56·2-80·0	2·4e-05*
	HN-PM	103	65·5	56·9-75·5		67·9	59·3-77·7	
	EXT	34	70·5	56·6-87·6		82·2	70·3-96·2	
	HN-nPM	40	72·5	59·9-87·7		82·5	71·5-94·2	
	UG-BP	48	77·1	66·1-90·0		83·3	73·4-94·6	
	ORB	42	83·1	72·4-95·3		95·1	88·7-100·0	
	UG-nBP	80	84·3	76·6-93·0		94·6	89·7-100·0	
**Tumor Site Group**								
	favourable	162	81·1	75·2-87·4	0·006*	91·6	87·4-96·1	5·5e-06*
	unfavourable	247	67·3	61·6-73·4		72·7	67·3-78·6	
**T-Status**								
	T1	208	81·9	76·8-87·4	6·6e-05*	90·1	86·1-94·3	1·7e-06*
	T2	201	63·3	56·9-70·3		69·9	63·8-76·7	
**Nodal Status**								
	N0	347	76·6	72·2-81·2	2·1e-05*	84·3	80·6-88·3	4·2e-07*
	N1	62	51·1	40·0-65·3		56·9	45·7-71·0	

[1]all data available, * significant, ukn. – unknown,
